# T-614, a novel immunomodulator, attenuates joint inflammation and articular damage in collagen-induced arthritis

**DOI:** 10.1186/ar2554

**Published:** 2008-11-19

**Authors:** Fang Du, Liang-jing Lü, Qiong Fu, Min Dai, Jia-lin Teng, Wei Fan, Shun-le Chen, Ping Ye, Nan Shen, Xin-fang Huang, Jie Qian, Chun-de Bao

**Affiliations:** 1 Shanghai Institute of Rheumatology, Renji Hospital, Shanghai Jiao Tong University School of Medicine, Shan Dong Middle Road, Shanghai 200001, PR China

## Abstract

**Introduction:**

T-614 is a novel oral antirheumatic agent for the treatment of rheumatoid arthritis. Whether it has immunomodulatory or disease-modifying properties and its mechanism of action are largely undetermined.

**Methods:**

Rats with collagen-induced arthritis (CIA) were treated with T-614 (5 and 20 mg/kg) daily. Animals receiving methotrexate (1 mg/kg every 3 days) and the nonsteroidal anti-inflammatory agent nimesulide (10 mg/kg per day) were used as controls. A combination therapy group was treated with both T-614(10 mg/kg per day) and methotrexate (1 mg/kg every 3 days). Hind paw swelling was evaluated and radiographic scores calculated. Serum cytokine levels were assessed by Bio-plex analysis. Quantitative PCR was used to evaluate expression of mRNA for interferon-γ, IL-4 and IL-17. Serum IL-17 and anti-type II collagen antibodies (total IgG, IgG_1_, IgG_2a_, IgG_2b _and IgM) were measured using ELISA.

**Results:**

Oral T-614 inhibited paw swelling and offered significant protection against arthritis-induced cartilage and bone erosion, comparable to the effects of methotrexate. CIA rats treated with T-614 exhibited decreases in both mRNA expression of IL-17 in peripheral blood mononuclear cells and lymph node cells, and circulating IL-17 in a dose-dependent manner. T-614 also reduced serum levels of tumor necrosis factor-α, IL-1β and IL-6. A synergistic effect was observed for the combination of methotrexate and T-614. In addition, T-614 (20 mg/kg per day) depressed production of anti-type II collagen antibodies and differentially affected levels of IgG_2a _subclasses *in vivo*, whereas IgM level was decreased without any change in the IgG_1 _level. Together, the findings presented here indicate that the novel agent T-614 has disease-modifying effects against experimental arthritis, as opposed to nimesulide.

**Conclusions:**

Our data suggested that T-614 is an effective disease-modifying agent that can prevent bone/cartilage destruction and inflammation in in CIA rats. Combination with methotrexate markedly enhances the therapeutic effect of T-614.

## Introduction

T-614 (N-[7-[(methanesulfonyl)amino]-4-oxo-6-phenoxy-4H-1-benzopyran-3-yl] formamide) is a novel immunomodulator. Previous research indicated that it could reduce immunoglobulin production by acting directly on B lymphocytes in both mice and humans, despite having no notable action on B-lymphocyte proliferation [1]. It also suppressed inflammatory cytokine production in cultured human synovial cells induced by tumor necrosis factor (TNF)-α by inhibiting the activity of nuclear factor-κB [2,3]. Reflecting laboratory findings, we observed significant improvements in rheumatoid arthritis (RA) in clinical trials [4]. The molecular mechanisms by which T-614 alters an ongoing immune response *in vivo *are not yet clear.

Rheumatoid arthritis (RA) is a complicated and treatment-refractory autoimmune disease that is characterized by a chronic inflammatory infiltrate of immune cells, in particular T cells, which represent approximately 40% of the synovial cellular infiltration and participate in a number of inflammatory and destructive events, such as synovial hyperplasia, pannus formation, cartilage and bone erosion, and joint malformation [5-8]. RA was previously considered to be a T-helper (Th)1-driven disease with a relative predominance of IFN-γ and lack of Th2 cytokines, leading to induction and persistence of disease. This was challenged by the demonstration that IL-17-producing T cells ('Th17' cells), and not IFN-γ CD4^+ ^effector T cells, are pathogenic in collagen-induced arthritis (CIA) [9,10]. Ligation of the IL-17 receptor, which is expressed on several cell types (including epithelial cells, endothelial cells, and fibroblasts), induces the secretion of IL-6, IL-8, granulocyte colony-stimulating factor, monocyte chemotactic protein-1, prostaglandin E_2_, TNF-α and IL-1β, as well as neutrophil chemotaxis and granulopoiesis [11-14]. IL-17 also induces the expression of matrix metalloproteinase-1 and -13 in RA synovial cells and osteoblasts [15,16], and induces the expression of RANKL (receptor activator of nuclear factor-κB ligand), which contributes to bone resorption [16].

Relative to other experimental arthritis models, CIA has been demonstrated to resemble human RA more closely in terms of clinical, histological and immunological features, as well as genetic linkage [17,18]. Dysregulated Th17 cell responses have been linked to the induction and progression of both CIA and RA. Local over-expression of IL-17 increases the severity of murine arthritis [19], and neutralizing anti-IL-17 antibody reduces the severity of arthritis [20]. IL-17-deficient mice have reduced incidence and severity of CIA [21]. An inhibitory effect on Th17 cells has been demonstrated for only a few drugs to date, including cyclosporine A [22] and entanercept [23].

In the present work we aimed to confirm the immunoregulatory effect of T-614, especially on Th17 cells, in CIA in rats. As a comparator drug, we evaluated the effect of methotrexate (MTX), one of the classical disease-modifying antirheumatic drugs (DMARDs) and the one that is most commonly used in clinical therapy, in CIA rats. We demonstrated that treatment of rats with T-614 dramatically suppressed disease progression, and markedly protected affected joints against cartilage destruction and bone erosion in a dose-dependent manner. Alleviation of Th17 cell differentiation and serum levels of IL-17 were first confirmed in CIA rats treated with T-614. The proinflammatory cytokines IL-6, TNF-α, and IL-β were decreased by treatment with T-614 (most significantly so for IL-6), contributing to the therapeutic effect of this agent. Even at low dose, T-614 in combination with MTX was able to inhibit the development of CIA completely. In addition, a comparison of T-614 with MTX suggested that T-614, but not MTX, inhibits the production of arthritogenic antibodies. In addition, nimesulide (an effective cyclo-oxygenase [COX]-2 inhibitor) depressed the edema and soft tissue swelling markedly in early disease, but it exhibited little inhibition of cartilage destruction and bone erosion. These findings indicate that T-614 exerts its immunoregulatory effect by skewing responses away from Th17, and by depression of antibody formation, which illustrate its unique character as a novel DMARD.

## Materials and methods

### Materials

T-614 was kindly provided by Simcere Pharmaceutical (Nanjing, China). Female Wistar rats (aged 6 to 7 weeks old, body weight 180 to 190 g) were purchased from the Laboratory Animal Services Center of the Shanghai Jiaotong University, School of Medicine (Shanghai, China). Animals were housed four per cage in rooms maintained at 20 ± 1°C with an alternating 12-hour light-dark cycle. Food and water were provided *ad libitum *throughout the experiments. Animals were acclimatized to their surroundings over 1 week to eliminate the effect of stress before initiation of the experiments. All of the experimental protocols involving animals and their care were approved by the Committee on Use of Human & Animal Subjects in Teaching and Research of the Shanghai Jiaotong University School of Medicine, and were carried out in accordance with the regulations of the Department of Health of Shanghai.

### Induction of CIA in rats and T-614 treatment

CIA was induced in female Wistar rats using a method described previously [24]. Briefly, rats were subcutaneously injected at the base of the tail with 200 μg bovine type II collagen (CII; Chondrex, Redmond, WA, USA) emulsified in complete Freund's adjuvant (Sigma, Redmond, WA, USA). On day 7 after primary immunization, all the rats were given an intradermal booster injection of 100 μg CII in incomplete Freund's adjuvant on the back (Sigma, Redmond, WA, USA). Onset of arthritis in ankle joints usually became visually apparent between days 10 and 12.

In the therapeutic treatment protocol for established CIA, all rats received treatment or vehicle (orally admininstered) from the day after onset of arthritis (day 12) until day 36 of the experiment. The rats received T-614 (daily dose 5 or 20 mg/kg body weight), nimesulide (Tocris Cookson, Ellisville, MO, USA; daily dose 10 mg/kg body weight), vehicle (0.5% CMC solution [vehicle] once daily), or MTX (Sigma, St. Louis, MO, USA; 1 mg/kg body weight every 3 days). Rats in the combination therapy group were administrated both MTX (1 mg/kg every 3 days) and T-614 (5 mg/kg per day).

### Evaluation of the development of arthritis

Clinical arthritis was observed daily and severity was assessed using a semiqualitative clinical score [25] as follows: 0 = normal, without any macroscopic signs of arthritis; 1 = mild, but definite redness and swelling of the ankle, or apparent redness and swelling limited to individual digits, regardless of the number of affected digits; 2 = moderate redness and swelling of the ankle; 3 = redness and swelling of the entire paw including digits; or 4 = maximally inflamed limb with involvement of multiple joints. In these studies, the maximum score was 8, which was the sum of scores from both hind paws of each animal.

### Radiographic assessments

Magnetic resonance imaging (MRI) was performed at day 21 with a 1.5 T magnetic resonance scanner Excite HD (General Electric Medical Systems, Milwaukee, WI, USA) using a 3-inch surface coil to obtain coronal short time inversion recovery (STIR) sequences. The acquisition parameters were as follows: repetition time 3,900 milliseconds, echo time 42.5 millisecond, field of view 60 mm, matrix 192 × 160 pixels, slice thickness 2 mm, interslice gap 0.2 mm, and scan time 2 minutes 18 seconds. In addition, coronal T1-weighted sequences were obtained (repetition time 540 milliseconds, echo time 16.1 milliseconds, field of view 60 mm, matrix 192 × 256 pixels, slice thickness 2 mm, interslice gap 0.2 mm, and scan time 2 minutes 18 seconds). MRI bone marrow edema was identified as hyperintense lesions on STIR sequences, with less clearly defined margins and intact trabecular structures [26].

High-resolution digital radiographs (24 kV, 40 mAs) of hind limbs were taken on all animals on day 36. Rats were given a score from 0 to 3 for each hind limb, with a summated maximum score of six based on the extent of soft tissue swelling, joint space narrowing, bone destruction, and periosteal new bone formation (0 = normal; 1 = soft tissue swelling only; 2 = soft tissue swelling and early erosions; and 3 = severe erosions).

Micro-computed tomography (CT) scans were done at the Shanghai Institute of Traumatology and Orthopaedics. Ankle bones were exposed to nondestructive three-dimensional imaging using a GE Medical Systems (London, Ontario) RS-9 *In Vivo *Micro-CT Scanner. The specimens were scanned on the micro-CT unit using the medium resolution (43.5 μm voxel dimensions in x, y, and z) scan mode. All scans were calibrated using samples of water, air, and a bone standard in order to allow consistent gray-level settings to be used when viewing the micro-CT images. A central sagittal section was generated for analysis from each mouse ankle bone image set using software available on the scanner console. Measurements of defection of the ankle bone were made using the software provided by the scanner manufacturer (MicroView, Waukesha, Wisconsin, USA).

### RNA extraction and real-time PCR analysis of IFN-γ, IL-4 and IL-17 expression

Total RNA was isolated from lymphocyte cells extracted with the TRIzol reagent (Invitrogen, Carlsbad, CA, USA) and reverse-transcribed using Sensiscript RT Kit (Fermentas, Burlington, Canada). mRNA expression for rat β-actin, IFN-γ, IL-4 and IL-17 was determined by real-time PCR using SYBR Green Master Mix (Applied Biosystems, Foster City, California, USA). The primers used are summarized in Table 1.

**Table 1:** Primers used

Molecule	Sense	Antisense
β-actin	5'-AGGCCAACCGTGAAAAGATG-3'	5'-ACCAGAGGCATAC AGGGACAA-3'
IFN-γ	5'-GAAAGACAACCAGGCCATCAG-3'	5'-TCATGAATGCATCCTTTTTTGC-3'
IL-4	5'-CCACGGAGAACGAG CTCATC-3'	5'-GAGAACCCCAGACTTGTTCTTCA-3'
IL-17	5'-GGGAAGTTGGACCACCACAT-3'	5'-TTCTCCACCCGGAAA GTGAA-3'

Thermocycler conditions included an initial holding at 50°C for 2 minutes, then 95°C for 10 minutes. This was followed by a two-step PCR program: 95°C for 15 seconds and 60°C for 60 seconds for 40 cycles. Data were collected and quantitatively analyzed on an ABI PRISM 7900 sequence detection system (Applied Biosystems). The β-actin gene was used as an endogenous control. The amount of gene expression was then calculated as the difference cycle threshold (ΔCT) between the CT value of the target gene and β-actin. ΔΔCT is the difference between the ΔCT values of the test sample and the control. Relative expression of target genes was calculated as 2^-ΔΔ*CT*^.

### Measurements of serum IL-17, TNF-α, IL-1β and IL-6 levels

Levels of the proinflammatory cytokines TNF-α, IL-1β and IL-6 in blood serum were measured up to day 28 for therapeutic treatments using commercially available Bio-plex kits (Research & Development, California, USA), in accordance with the manufacturers' recommendations. Serum specimens for IL-17 detection were analyzed by ELISA. Microtiter plates were coated with antibody of IL-17 (Santa Cruz Biotechnology, Santa Cruz, CA, USA) overnight at 4°C, and then blocked (0.01 mol/l phosphate-buffered saline [PBS]/0.05% bovine serum albumin; this solution was used for all further dilutions) for 2 hours at 37°C. Rat sera were diluted with PBS at 1:20 and added in duplicate wells. Plates were incubated for 2 hours, and subsequently horseradish peroxidase-conjugated goat anti-rat antibody were added and incubated for 45 minutes. At every step, plates were washed three times with 0.01 mol/l PBS containing 0.05% Tween-20. 3,3',5,5'-Tetramethylbenzidine were used for color development. Absorbance (mU) was read at 450 nm and values were expressed as mean ± standard error of the mean (Bio-Rad Laboratories, Hercules, CA, USA).

### Measurement of type II collagen antibodies

Antibody titers to type II collagen were assayed by ELISA. Nunc Maxisorb plates were coated with 100 μl of bovine nasal collagen II (5 μg/ml in PBS) overnight at 4°C, and then blocked (0.01 mol/l PBS/0.05% bovine serum albumin; this solution was used for all further dilutions) for 2 hours at 37°C. Serum samples were diluted 1:1,000, and 100 μl was added to the coated 96-well plate and incubated at 37°C for 2 hours, followed by a 2-hour incubation with a horseradish peroxidase-linked goat anti-rat IgG antibody (KPL, Gaithersburg, MD, USA) and mouse anti-rat IgG_1_, IgG_2a_, IgG_2b _and IgM antibody (Southern Biotech, Birmingham, AL, USA). At every step, plates were washed three times with 0.01 mol/l PBS containing 0.05% Tween 20. Absorbance (mU) was read at 450 nm and values were expressed as mean ± standard error of the mean. Optical density was measured using Microplate computer software (Bio-Rad Laboratories).

### Data analysis

Significant changes in clinical arthritis as a result of drug treatment were determined using a dynamic modeling approach, assuming a linear fit for the slope of arthritis progression for each individual animal (SAS Institute, Inc., Cary, NC, USA). Significant differences in serum cytokines and antibody levels were assessed using the Student's *t*-test, and *P *< 0.05 was considered statistically significant. The clinical and radiological score was analyzed using nonparametric analysis; Mann-Whitney test was used when two groups were compared. To test for differences in trends during the study among study groups, we used Kruskal-Wallis method followed by Dunn's test to evaluate differences in each of the study groups from days 12 to day 30, adjusted to baseline values at day 12.

## Results

### Decrease in the development of collagen induced arthritis rats treated with T-614

**Figure 1:**
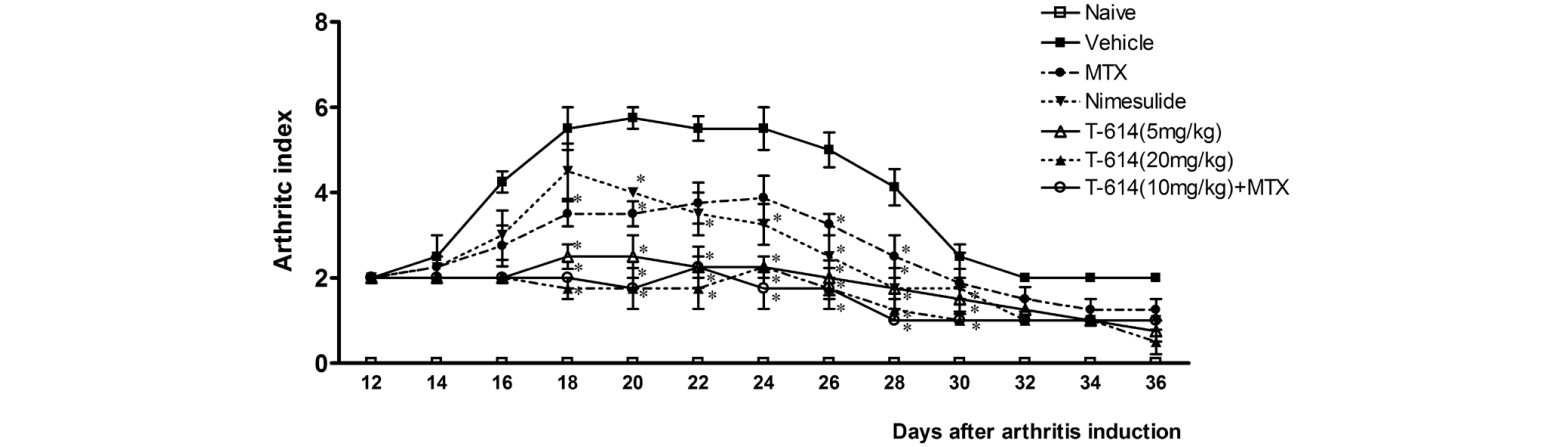
Effects of therapeutic treatment with T-614 on disease progression in rats with established CIA. Rats were orally treated daily with T-614 at 5 mg/kg per day or 20 mg/kg per day; MTX at 1 mg/kg every 3 days; nimesulide at 10 mg/kg per day; T-614 at 10 mg/kg per day and MTX at 1 mg/kg every 3 days; or vehicle. Treatment began on day 12 after immunization with type II collagen until day 36. Data are expressed as mean ± standard error of the mean (n = 5 to 7). *P < 0.05, **P < 0.01, versus day 12 or the vehicle-treated rats. CIA, collagen-induced arthritis; MTX, methotrexate.

The CIA model is characterized by aggressive synovitis, extensive pannus formation, cartilage degradation, and focal bone erosion. We investigated whether the protective activity of T-614 was mediated through a decrease in the severity of all of these clinical indices, or whether the activity of T-614 affected only specific pathogenetic processes. As shown in Figure 1, even after the onset of arthritis, T-614 (5 and 20 mg/kg per day) markedly reduced arthritic scores in the arthritic rats in a dose-dependent manner, as compared with the vehicle-treated arthritic rats.

Progression of disease was indicated by increased edema and erythema of one or both ankle joints, followed by involvement of the metatarsal and interphalangeal joints. Fully developed arthritis, including red and swollen paws, was observed 8 to 10 days after onset of inflammation. The clinical score in the vehicle-treated group reached a peak approximately 20 days after the first immunization (maximum arthritis score of 5.75 ± 0.5; *P *< 0.01, versus day 12). Treatment with MTX (1 mg/kg every 3 days) was efficacious and resulted in a delayed peak (day 24) and also reduced clinical arthritis significantly at day 20 (clinical score 3.5 ± 0.57; *P *< 0.0286, versus vehicle). Signs of moderate arthritis were observed in rats treated with a low dose of T-614 (5 mg/kg), which became most severe at day 18 (maximal clinical score = 2.5 ± 0.6; *P *= 0.0286, versus day 12) and improved significantly at day 20 (clinical score = 2.5 ± 1; *P *= 0.0286, versus vehicle). The high-dose T-614 (20 mg/kg per day) and combination treatments almost completely suppressed progression; maximal clinical scores in these rats were 1.75 ± 0.9 at day 24 and 1.73 ± 0.8 at day 22, respectively (*P *> 0.05, versus day 12). The clinical score in the high-dose T-614 and combined treatment groups was found to be statistically significantly lower than that in the control group at day 20; the maximal clinical scores in these two groups were 1.75 ± 0.975 and 1.75 ± 0.79, respectively (*P *< 0.05, versus vehicle). Measurements of paw thickness and paw circumference were consistent with clinical scores (data not shown).

### Decrease in the severity of inflammation in collagen induced arthritis rats treated with T-614

**Figure 2:**
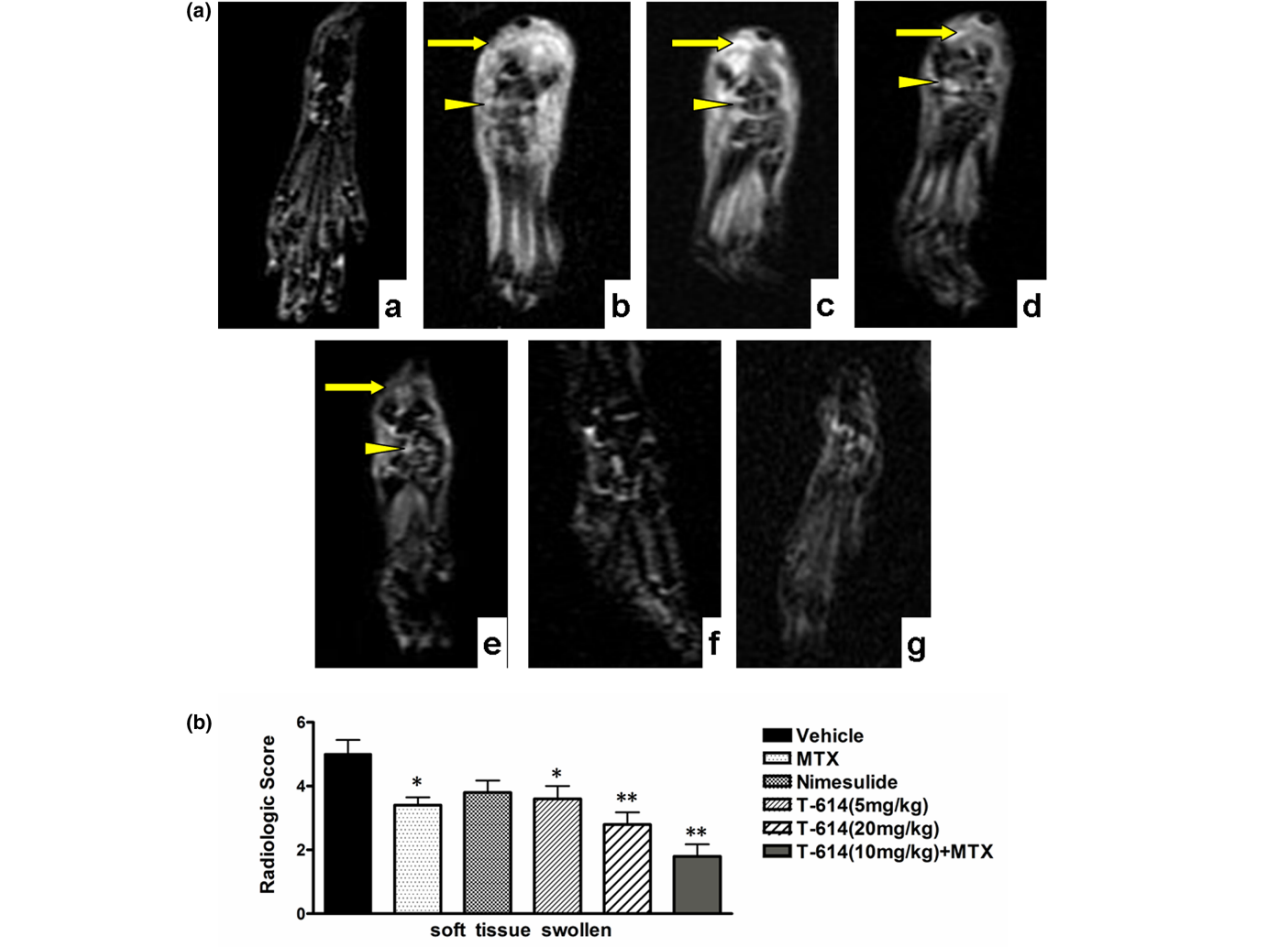
Effects of therapeutic treatment with T-614 on inflammation in the CIA rats. **(a) **STIR magnetic resonance images of hind paws from CIA rats. The presence of soft tissue swelling (yellow arrow) and localization of bone marrow edema (yellow triangle) are highlighted. Neither paw swelling nor bone marrow edema was seen in normal rats (subpanel a). Severe soft tissue swelling and bone erosion were seen in CIA rats treated with vehicle (subpanel b). Similar damage was observed in rats treated with nimesulide (subpanel d), but much less damage was seen in rats treated with MTX (subpanel c), T-614 (subpanels e and f), and combination treatment with T-614 and MTX (subpanel g). **(b) **Magnetic resonance imaging score of soft tissue swelling in treated CIA rats. Data are expressed as mean ± standard error of the mean (n = 3 to 5). *P < 0.05, **P < 0.01, versus vehicle-treated arthritic rats. CIA, collagen-induced arthritis; MTX, methotrexate.

The morphologic changes in the joint architecture of CIA rats were further assessed using MRI, 21 days after the first immunization. MRI soft tissue swelling is defined based on penetration of subcutaneous soft tissues and bone marrow on the T1-weighted image within normal hyperintense subcutaneous soft tissues and bone marrow. This corresponds to findings on the STIR image (Figure 2a), in which the damage can be seen as a clearly demarcated zone of hyperintense signal within normal hypointense area at this site (arrowhead).

Joints of naïve (non-CIA) rats exhibited intact joint architecture. The talus, phalanges, talocalcaneal joints, talonavicular articulations and cuneonavicular joints were well defined. Joints from the vehicle-treated CIA group exhibited significant damage as well as swelling of soft tissues and marked bone marrow edema. T-614 had a dose-related efficacy. Joints from rats treated with MTX (1 mg/kg every 3 days) or nimesulide (10 mg/kg per day) also exhibited moderate damage, whereas nimesulide was associated with much less inhibition of bone marrow edema. Joints from the T-614 (20 mg/kg per day) alone and combination therapy group exhibited significant inhibition of damage, which closely resembled the joints from the naïve rats. As shown in Figure 2b, the mean MRI soft tissue swelling scores in vehicle-treated (5 ± 0.45) and nimesulide-treated rats (3.8 ± 0.37; *P *= 0.96, versus vehicle) were significantly higher than those in rats treated with low-dose T-614 (3.6 ± 0.4; *P *= 0.0479, versus vehicle), high-dose T-614 (2.8 ± 0.37; *P *= 0.0159 versus vehicle), and MTX (3.4 ± 0.25; *P *= 0.0318, versus vehicle). The hind paws of CIA rats receiving combined treatment with MTX and T-614 exhibited complete protection, with the lowest soft tissue swelling scores (1.8 ± 0.38; *P *= 0.0079, versus vehicle).

### Preservation of the structural integrity of affected joints by T-614 treatment

**Figure 3:**
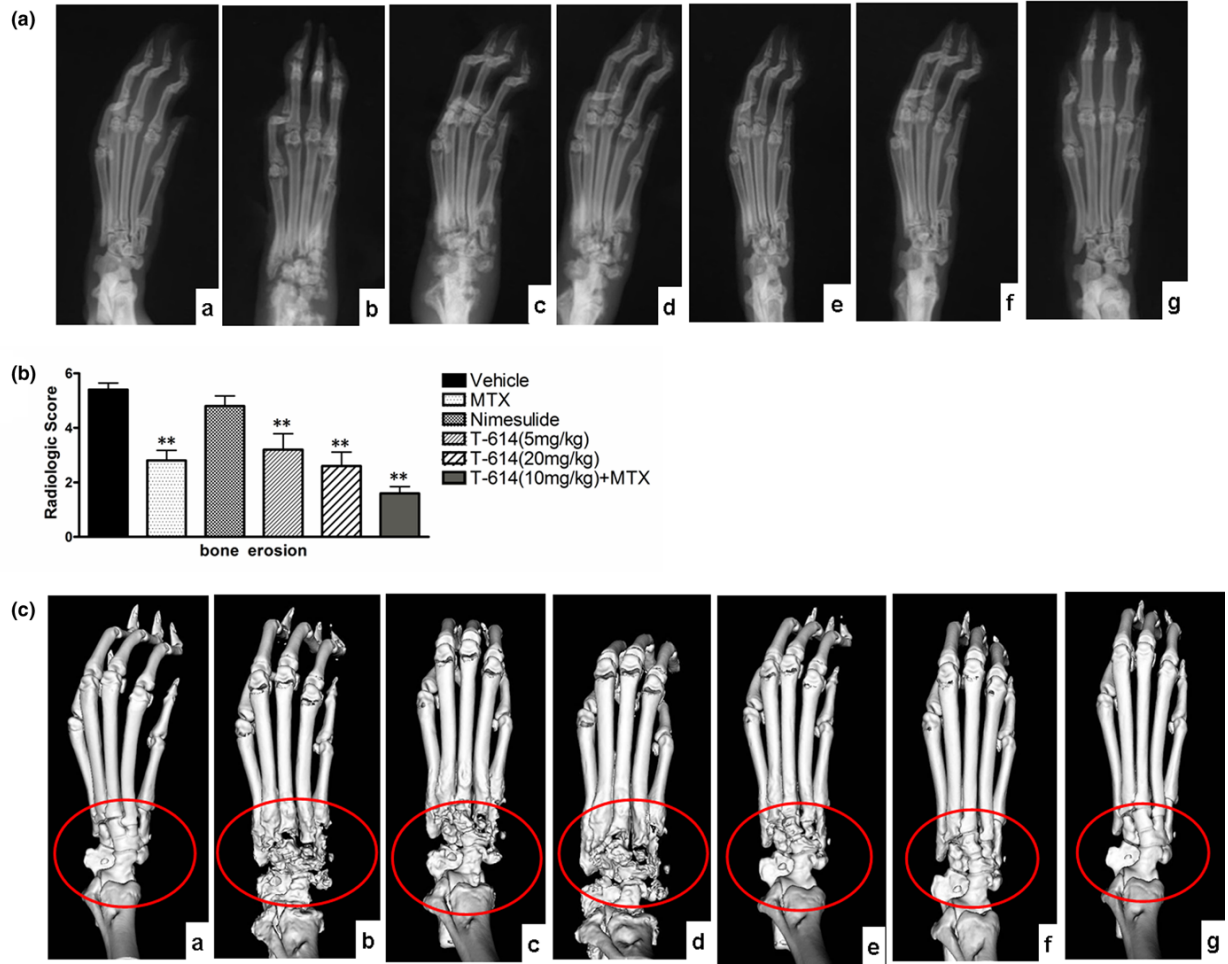
Effects of treatment with T-614 on structural integrity in CIA rats. **(a) **Macroradiographs of rat hind paws. Neither paw swelling nor joint damage was observed in normal rats (subpanel a). Severe bone matrix resorption and erosion were seen in CIA rats treated with vehicle (subpanel b). Similar damage was observed in rats treated with nimesulide (subpanel d), but the damage was much less in rats treated with MTX (subpanel c), T-614 (subpanels e and f), and both of them (subpanel g). **(b) **Radiologic score of bone erosion in treated CIA rats. Data are expressed as mean ± standard error of the mean (n = 3 to 5). *P < 0.05, **P < 0.01 versus the vehicle-treated rats. **(c) **All images were obtained using a RS-9 in Vivo Micro-CT. Neither joint damage nor bone loss was seen in normal rats (subpanel a). Severe bone matrix resorption, erosion joint, and bone loss were seen in CIA rats treated with vehicle (subpanel b). Similar bone loss was seen in rats treated with nimesulide (subpanel d) but this was much less in rats treated with MTX (subpanel c), T-614 (subpanels e and f), and the combination of T-614 and MTX (subpanel g). CIA, collagen-induced arthritis; MTX, methotrexate.

The hind paws were further examined by radiography and micro-CT at day 36. Radiographic severity of joint destruction in the ankle joints of rats treated with T-614 was markedly reduced compared with those in the MTX-treated and nimesulide-treated CIA rats. Representative radiographs of the hind paws from vehicle, MTX, nimesulide and T-614 rats are shown in Figure 3a. Radiological analysis revealed severe bone erosion in the joints of CIA rats, as shown in Figure 3b. The mean bone erosion scores in vehicle (4.4 ± 0.25) and nimesulide rats (4.8 ± 0.38; *P *= 0.309, versus vehicle) were significantly higher than those in rats receiving low-dose T-614 (3.2 ± 0.58; *P *= 0.015, versus vehicle), high-dose T-614 (2.6 ± 0.5; *P *= 0.007, versus vehicle), and MTX (2.8 ± 0.37; *P *= 0.009, versus vehicle). The hind paws of CIA rats receiving combined treatment with MTX and T-614 exhibited complete protection, with the lowest scores for bone erosion (1.6 ± 0.24; *P *= 0.007, versus vehicle). The data also indicate that MTX markedly inhibited the bone erosion of the arthritic joints, as did T-614 (5 mg/kg per day), but not the soft tissue swelling.

We further investigated the effect of T-614 treatment on structural preservation of hind joints in rats with established disease by three-dimensional micro-CT imaging, which permits noninvasive visualization of pathologic joint changes (Figure 3c). Images of a naïve rat joint revealed intact joint architecture as well as normal bone surfaces. The various bones that constitute the joint, namely the distal tibia/fibula, talus and calcaneus, were clearly resolved. The joint from the CIA rats treated with vehicle and nimesulide exhibited marked erosion of several bone surfaces, especially at the junction of the distal tibia and fibula and along the length of the calcaneus. Degenerative changes were also visible on the talus. Compared with CIA rats treated with MTX and low-dose T-614, those CIA rats treated with either high dose (20 mg/kg per day) T-614 alone or 10 mg/kg per day T-614 combined with MTX resulted in much more significant protection against bone destruction, preservation of the architecture of the affected hind joints, and protection against degenerative changes. Isolated regions of bone erosion could be visualized, but the integrity of the joint architecture was clearly preserved.

### Skewing of responses away from Th17 in CIA by T-614 treatment

**Figure 4:**
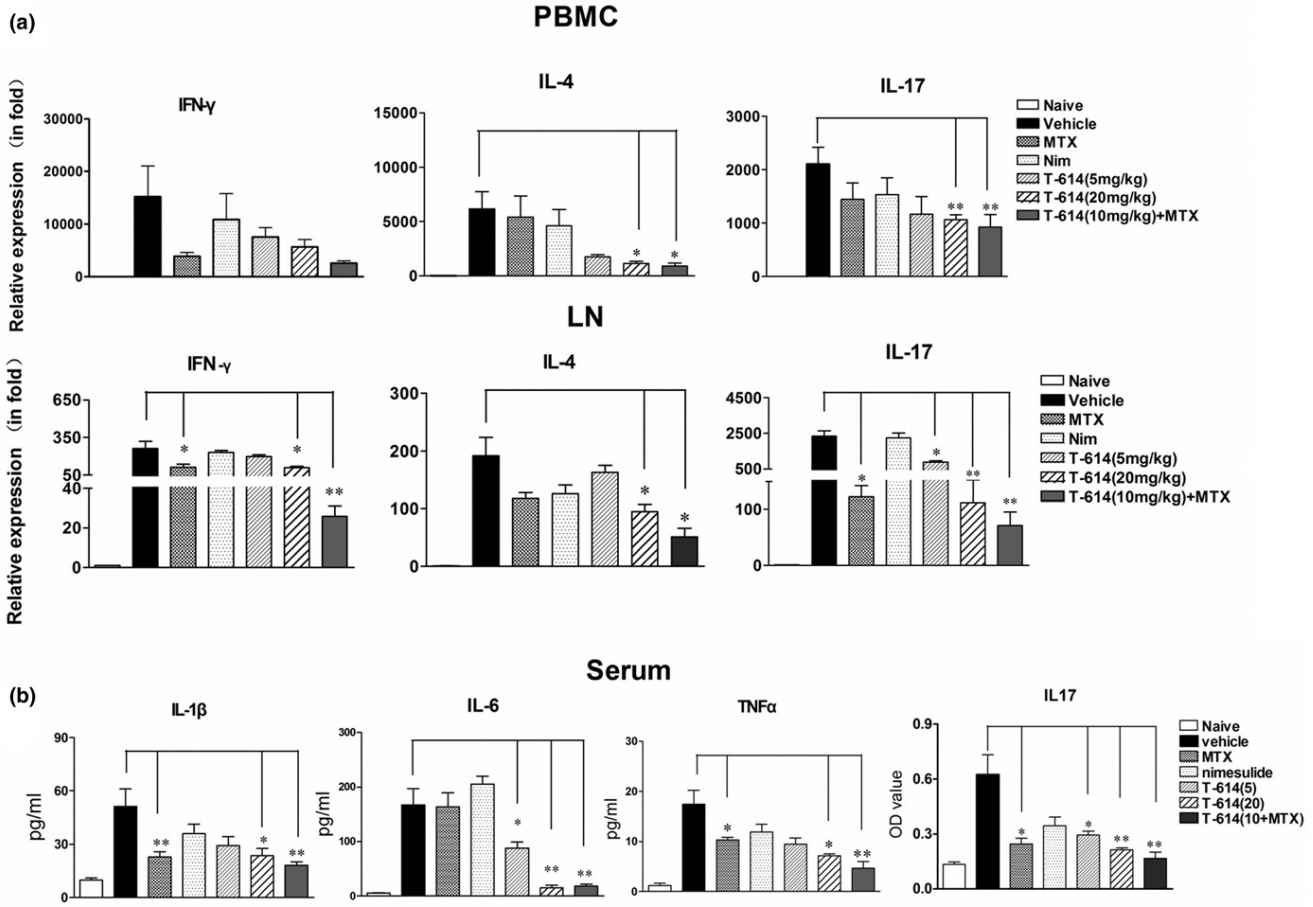
Effects of T-614 on cytokine levels in CIA rats. Rats were orally treated with different doses of T-614, nimesulide, MTX and vehicle, beginning on day 12 after the immunization with CIA until day 36. **(a) **Effects of T-614 on mRNA levels of IFN-γ, IL-4 and IL-17 in lymph node and PBMCs from CIA rats. **(b) **Effects of T-614 on serum levels of TNF-α, IL-1β, IL-6 and IL-17 in the CIA rats. CIA, collagen-induced arthritis; IFN, interferon; IL, interleukin; LN, lymph node; MTX, methotrexate; PBMC, peripheral blood mononuclear cell.

Expression levels of transcripts for T-cell differentiation related genes, namely IFN-γ, IL-4 and IL-17, in the inguinal lymph node and peripheral blood mononuclear cells (PBMCs) were analyzed on day 21 after immunization with CIA (Figure 4a). levels of IL-4 and IL-17 decreased sharply in the PBMCs from high-dose T-614 and combination treated rats. In particular, T-614 inhibited the elevated IL-17 expression in inguinal lymph node cells in a dose-dependent manner. IFN-γ and IL-17 mRNA levels, but not those of IL-4, decreased significantly in lymph nodes of rats treated with MTX. No treatment was able to depress the elevated IFN-γ expression in PBMCs.

Levels of proinflammatory cytokines TNF-α, IL-1β, and IL-6 in blood serum were analyzed using a multiplex immunoassay on day 28 after immunization with CIA. IL-17 level was determinated by ELISA analysis. Consistent with the joint swelling, TNF-α, IL-1β, IL-6, and IL-17 in the vehicle-treated CIA rats were systemically over-produced in serum. The elevated IL-6 and IL-17 levels in rats treated with T-614 were decreased in a dose-dependent manner and correlated positively with the degree of joint swelling in individual animals. T-614 at the dose of 20 mg/kg only, but not at 5 mg/kg, significantly reduced serum levels of TNF-α and IL-1β (Figure 4b).

### Disease attenuation is also partly attributable to inhibition of humoral collagen-specific immunity

**Figure 5:**
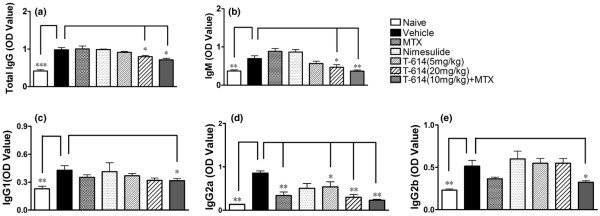
Effects of T-614 on serum IgG levels in CIA rats. Serum was collected on day 36. Anti-CII (total IgG, IgM, IgG_1_, IgG_2a_, and IgG_2b_) levels increased as disease progressed. Combination therapy reduced the total anti-CII antibody level significantly, as well as levels of IgM, IgG_1_, IgG_2a_, and IgG_2b_. High dose T-614(20 mg/kg per day) also decreased levels of total IgG, IgM and IgG_2a_, whereas low-dose of T-614 (5 mg/kg per day) or MTX (1 mg/kg every 3 days) had an effect only on IgG_2a _level. Data are expressed as mean ± standard error of the mean (n = 5 to 7). *P < 0.05, versus the vehicle-treated rats.

T-614, but not MTX, strongly inhibited the increase in CII antibody. To determine the effect of T-614 on immunoglobulin subclasses, the total serum levels of IgM, IgG_1_, IgG_2a_, and IgG_2b _subclasses were quantified. As shown in Figure 5, there was no significant difference in total IgG-CII antibody (*P *< 0.05) between MTX, nimesulide and low-dose T-614 groups compared with the vehicle control. Anti-CII antibody levels in sera from rats treated with combination therapy were markly decreased, as were levels of IgG_1_, IgG_2a_, IgG_2b _and IgM. High-dose T-614 (20 mg/kg per day) also decreased levels of total IgG, IgG_2a _and IgM, whereas low-dose T-614 (5 mg/kg per day) affected only the level of IgG_2a_. Moreover, the IgG_2a _level was also decreased in the MTX group.

## Discussion

RA is a complicated and treatment-refractory autoimmune disease, with complex pathogenesis and involving pathological changes in multiple targets [5,27,28]. The joint targeted effector mechanism of the classical model is probably quite complex, involving T-cell stimulation of synovial cells, T-cell independent mesenchymal activation, and an arthritogenic effect in which antibodies bind to cartilage. The proinflammatory cytokines, mainly TNF-α, IL-1β and IL-6, are considered powerful targets in the treatment of RA [29-31]. The new biologic agents, despite their substantial efficacy and ability to bring about clinical improvement, are expensive and cause hypersensitivity to medications and infections [32-34]. Because long-term experience with anti-TNF therapy is limited, the potential long-term risks, particularly of developing lymphomas, remains an issue [30]. Until these concerns are fully addressed, nonbiologic DMARDs will probably remain the preferred initial treatments for RA [35,36]. Because of its multi-suppressive properties, T-614 is expected to be applied in treatment of RA independently or combined with other DMARDs such as MTX, an analog of folic acid and of aminopterin. MTX was therefore included as a standard control in our studies because of its dramatic effects on arthritis in rat models [37]. Nimesulide, an effective COX-2 inhibitor, was also tested to identify the role played by nonsteroidal anti-inflammatory drugs in the development of CIA.

Clearly, both T-614 and MTX efficiently suppress the CIA model after the onset of arthritis. Soft tissue swelling and bone marrow edema in early CIA were measured, and paw architecture was examined using MRI [38]. Compared with the clinical score data, MRI results provided more objective and detailed information. Our findings indicate that low-dose T-614 (5 mg/kg per day) suppressed autoimmune responses to a degree similar to that with MTX (1 mg/kg every 3 days), whereas high-dose T-614 (20 mg/kg per day) almost completely inhibited the inflammation and bone marrow edema of CIA. When combined with MTX, T-614 (10 mg/kg per day) was able to effect complete control of the disease process. Inhibition the activity of COX-2 by nimesulide also depressed the edema of CIA paws effectively, whereas the bone marrow edema continued to progress.

The role played by T cells in RA has been highlighted by IL-17, a T-cell derived proinflammatory cytokine that has been implicated in joint inflammation and destruction [8,39-41].

Because the treatment was started after the onset of arthritis, it did not affect immune priming following immunization or the earliest inflammatory events with synovial hyperplasia, infiltration of inflammatory cells and differentiation of collagen II-specific T cells. Our data demonstrate the powerful inhibitory and dose-dependent effect of T-614 on IL-17 levels in local lymph nodes. The immunomodulatory effect of T-614 is not clear but it may partly depend on its inhibition of nuclear factor-κB or other cell signaling pathways [42]. Real-time PCR is sensitive and allows immediate assessment of mRNA expression, but it still differs from the protein level. There remains much work to be done to identify the specific cytokine-secreting T cells and confirm their differentiation. Bone preservation appeared to be one of the main benefits of IL-17 inhibition, and this feature was reflected in the ankle bone volumes calculated quantitatively by micro-CT imaging. Rats receiving T-614 at 5 mg/kg per day exhibited significantly less bone destruction (*P *< 0.05), as measured by total bone volume, compared with vehicle-treated arthritic controls. The bone volumes of rats receiving T-614 at 20 mg/kg per day and T-614 combined with MTX remained almost intact. The findings support the view that T-614 can protect the joints from damage in an inflammatory environment, in concert with MTX.

Proinflammatory cytokines TNF-α, IL-1β, and IL-6 help to propagate the extension of a local or systemic inflammatory process. Similar to the IL-17 levels in serum, markedly low serum levels of IL-6 were also observed in CIA rats treated with T-614, even at the dose of 5 mg/kg per day. Only MTX, high-dose T-614 (20 mg/kg per day) and not low-dose T-614 (5 mg/kg per day), and combination treatment significantly reduced serum levels of TNF-α and IL-1β. Recent studies have shown that IL-6, in combination with transforming growth factor-β, inhibits the generation of FoxP3-expressing T-regulatory cells and induces the generation of Th17 cells [43]. Th1, Th2, and Th17 cells develop from naïve T cells; in contrast, the generation of T-regulatory cells and Th17 cells occurs via alternative pathways, and they are selected according to the presence or absence of IL-6, a pleiotropic cytokine that plays important roles in the regulation of the immune response, inflammation, and hematopoiesis. Decreased IL-6 production could contribute to the attenuation of Th17 responses, which may also explain the therapeutic effect of T-614. IL-6 also induces activated B cells to differentiate into antibody-producing cells [44] and promotes the production of vascular endothelial growth factor, which plays an important role in angiogenesis [45]. Furthermore, in terms of bone metabolism, IL-6 induces osteoclast differentiation in the presence of soluble IL-6 receptor, thereby contributing to joint destruction and osteoporosis [46]. IL-17 significantly induces the synthesis of IL-6 by synoviocytes and macrophages. A positive feedback loop initiates and accelerates the progression of CIA. Modulation of inflammatory cytokines and IL-17 by T-614 suggests its potential therapeutic value in the treatment of other inflammatory diseases, such as ankylosing spondylitis and psoriatic arthritis.

During the development of CIA, increasing levels of anti-CII antibodies bind to the collagen of the articular cartilage, activate the complement system and initiate tissue damage; this indicates that there is T-B cell cooperation and activation *in vivo *[47,48]. More interestingly, T-614 not only suppressed CII antibody levels but also differentially modulated immunoglobulin subclass levels; these effects suggest that it may be useful for the treatment of lupus or other autoimmune disorders. Similar effects were seen in the combination therapy group, indicating that there is synergy between T-614 and MTX. Low-dose T-614 and MTX also had an effect on the level of IgG_2a _antibody, indicating that they may operate through T-cell associated antibodies in the CIA model. Because IgG_2a _is the most potent activator of the classical complement cascade and Fc receptor bearing macrophages, the present findings add further support to the inhibitory mechanism of T-614 and the pathogenic role of IgG_2a _in rat CIA [49].

To summarize, T-614 – a novel immunomodulatory drug – appears to protect the joints from inflammation injury and osteoclastic bone resorption through skewing the response from primarily a Th17-driven one, and it does so to a greater degree in combination with MTX. These findings suggest that T-614 is a new candidate for use in combination therapy, which is increasingly being applied to the treatment of RA and other Th17-associated inflammatory autoimmune diseases.

## Conclusion

In the present experiments, T-614 significantly prevented bone/cartilage destruction and inflammation in CIA. Furthermore, combination with MTX enhanced the therapeutic effect of T-614.

## Abbreviations

CIA: collagen-induced arthritis; CII: type II collagen; CT: computed tomography; ΔCT: difference cycle threshold; DMARD: disease-modifying antirheumatic drug; ELISA: enzyme-linked immunosorbent assay; IFN: interferon; IL: interleukin; MRI: magnetic resonance imaging; MTX: methotrexate; PBMC: peripheral blood mononuclear cell; PCR: polymerase chain reaction; RA: rheumatoid arthritis; Th: T-helper; TNF: tumor necrosis factor; STIR: short time inversion recovery

## Competing interests

The authors declare that they have no competing interests.

## Authors' contributions

CB designed and conceived the study. FD conducted the experimental work and drafted the manuscript. SC participated in the design of the study. LL performed the statistical analysis. JT, MD, WF, PY, NS, XH and JQ helped with some experimental work. All authors read and approved the final manuscript.
